# Clinical, hematologic and molecular variability of sickle cell-β thalassemia in western India

**DOI:** 10.4103/0971-6866.73410

**Published:** 2010

**Authors:** Malay B. Mukherjee, Anita H. Nadkarni, Ajit C. Gorakshakar, Kanjaksha Ghosh, Dipika Mohanty, Roshan B. Colah

**Affiliations:** Department of Hemato-Genetics, National Institute of Immunohaematology (ICMR), Mumbai, India

**Keywords:** HbS- β thalassemia, clinical, mutations, framework, India

## Abstract

**BACKGROUND:**

Sickle cell-β thalassemia (HbS-β thalassemia) is a sickling disorder of varying severity, which results from compound heterozygosity for sickle cell trait and β thalassemia trait. The present study was undertaken to determine the genetic factors responsible for the clinical variability of HbS-β thalassemia patients from western India.

**MATERIALS AND METHODS:**

Twenty-one HbS-β thalassemia cases with variable clinical manifestations were investigated. The α and β globin gene clusters were studied by molecular analysis.

**RESULTS:**

Thirteen patients showed milder clinical presentation as against eight patients who had severe clinical manifestations. Four β thalassemia mutations were identified: IVS 1-5 (G→C), codon 15 (G→A), codon 30 (G→C) and codon 8/9 (+G). α thalassemia and XmnI polymorphism in homozygous condition (+/+) were found to be common among the milder cases. The β^S^ chromosomes were linked to the typical Arab-Indian haplotype (#31). Framework (FW) linkage studies showed that four β thalassemia mutations were associated with different β globin gene frameworks. Linkage of codon 15 (G→A) mutation to FW2 is being observed for the first time.

**CONCLUSION:**

The phenotypic expression of HbS-β thalassemia is not uniformly mild and α thalassemia and XmnI polymorphism in homozygous condition (+/+) are additional genetic factors modulating the severity of the disease in the Indian subcontinent.

## Introduction

The β^s^ mutation is one of the most common single gene mutations in man and has a widespread geographic distribution including most of Africa, the Middle East, India and parts of Mediterranean.[1] In India, the frequency of the β^s^ gene reaches as high as 40% especially in the tribal groups,[2] whereas the incidence of the β thalassemia gene is around 3-4% in the general population.[3] Hence, the occurrence of HbS- β thalassemia due to inheritance of both the defects is expected to occur. The clinical features of HbS- β thalassemia are extremely variable, ranging from a completely asymptomatic state to a severe disorder similar to homozygous sickle cell disease.[4] This heterogeneity is likely to be due to the presence of different β thalassemia alleles or interaction with modulating genetic factors like associated α thalassemia and/or a gene for raised HbF production (XmnI polymorphism).

Our population survey in Maharashtra and Gujarat from western India revealed a high prevalence of the β^s^ gene in different population groups.[5] During this study, 21 sickle cell- β thalassemia cases (7 tribals and 14 non tribals) in the age group of 3-25 years were identified using solubility test, hemoglobin electrophoresis and measurement of HbA_2_ levels by elution after electrophoresis. Further, these cases were confirmed by family studies. Here, we report the clinical and hematologic presentation and molecular pathology of these HbS-β thalassemia cases from western India.

## Materials and Methods

Information on age at presentation, requirement of blood transfusions, frequency of vaso-occlusive crisis per year, liver and spleen size were recorded. Ten milliliters of blood was collected in ethylenediaminetetraacetic acid (EDTA) after informed consent was obtained from all subjects. Blood samples for hematologic evaluation were collected at least 30 days after the last transfusion. RBC indices were measured on an automated blood cell counter (Syxmex K-1000). Hemoglobin electrophoresis and quantitation of HbA_2_ was done using alkaline cellulose acetate electrophoresis at pH 8.9 and elution.[6] The HbF level was quantified by the alkali denaturation method of Singer *et al*.[7] Molecular analysis was carried out in 19 HbS-β thalassemia cases. DNA was isolated from peripheral blood leukocytes using the standard phenol-chloroform method. The HbS and β thalassemia mutations were characterized by reverse dot blot hybridization,[8] while α globin genotype, β^s^ haplotype and the presence of a C→T mutation at position -158 of the G_γ_gene (XmnI polymorphism) were determined as described earlier.[9] The framework (FW) analysis was done by Denaturing gradient gel electrophoresis (DGGE) analysis.[10]

## Results

The clinical, hematologic and molecular data of these cases are summarized in Table 1. The age at presentation varied from 6 months to 18 years. Seven HbS- β thalassemia cases in the tribal group had a milder clinical presentation, whereas 8 of the 14 cases (57.2%) in the nontribal group had severe clinical manifestations with a history of vaso-occlusive crisis (3-8 per year) in the form of acute pain in the joints, abdomen, bones and chest and they were also dependent on regular blood transfusions. Some of these patients had infections, usually in the form of high grade fever and also required hospitalization for their painful crisis. Although hepatosplenomegaly was observed in both mild and severe cases, however, splenomegaly was more common in severe cases (87.5%) as compared to milder cases (53.8%).

**Table 1:** Clinical, hematologic and molecular data of HbS-β thalassemia cases from western India

Tribal/nontribal	Age/sex	Age at presentation	VOC (per year)	Transfusions (per year)	Liver (cm)	Spleen (cm)	RBC (×10^6^/μl)	Hb (g/dl)	MCV (fl)	MCH (pg)	HbA2 (%)	HbS (%)	HbF (%)	XmnI	βThal. mut	α Genotype
Tribal*	7/F	7 years	1	0	1	1	3.2	7.8	73.0	24.2	6.2	77.7	22.1	+/+	Cd 15 (G→A)	-α/-α
Tribal*	21/M	20 years	1	0	1	1	5.0	9.6	61.0	19.0	4.4	88.9	18.7	+/-	Cd 15 (G→A)	-α/αα
Tribal*	14/F	10 years	0	0	0	0	5.3	8.6	61.0	19.8	5.0	79.1	15.8	+/-	Cd 15 (G→A)	-α/αα
Tribal*	18/M	AS	0	0	0	2	4.0	8.8	69.0	21.5	4.7	75.7	19.0	+/-	Cd 15 (G→A)	-α/αα
Tribal*	16/F	AS	0	0	0	1	4.4	8.2	71.0	22.4	5.0	78.1	19.4	+/-	Cd 15 (G→A)	-α/-α
Tribal*	12/F	8 years	1	2	1	1	4.8	9.2	65.0	24.1	4.9	78.4	12.1	NT	NT	NT
Tribal*	13/M	AS	0	0	1	0	4.5	8.9	70.0	23.2	4.8	72.3	18.8	NT	NT	NT
Nontribal*	8/F	8 years	0	0	1	2	3.6	11.5	74.0	22.6	4.9	54.7	31.6	+/+	IVS 1-5 (G→C)	αα/αα
Nontribal**	19/M	AS	0	0	0	0	4.3	10.9	70.0	25.3	4.3	67.1	23.6	+/+	Cd 15 (G→A)	αα/αα
Nontribal**	25/F	8 years	1	3	0	0	3.7	10.4	71.0	27.7	4.2	34.6	12.7	+/-	IVS 1-5 (G→C)	-α/αα
Nontribal**	10/F	AS	0	0	0	0	3.5	7.2	63.6	20.5	5.6	60.5	20.5	+/+	IVS 1-5 (G→C)	-α/-α
Nontribal*	17/F	AS	0	0	0	0	3.6	9.6	77.4	26.4	6.6	61.3	18.6	+/+	Cd 30 (G→C)	-α/αα
Nontribal*	19/M	AS	0	0	0	2	4.3	10.9	70.0	25.3	4.4	67.1	23.6	+/+	IVS 1-5 (G→C)	-α/-α
Nontribal**	12/F	1 year	3	12	1	3	3.3	10.0	68.0	21.3	3.9	50.0	10.0	+/-	IVS 1-5 (G→C)	-α/αα
Nontribal**	3/M	1 year	8	10	0	2	2.8	7.1	66.0	20.7	3.9	60.0	3.0	+/-	IVS 1-5 (G→C)	αα/αα
Nontribal**	24/M	3 years	6	11	3	2	3.5	8.6	71.8	24.2	4.3	40.1	5.6	+/-	IVS 1-5 (G→C)	αα/αα
Nontribal**	4/F	6 months	8	12	1	2	3.2	9.3	62.1	16.5	5.2	40.3	5.6	+/-	Cd 30 (G→C)	αα/αα
Nontribal**	10/M	1 year	4	15	0	0	4.5	11.9	73.4	25.9	5.1	30.1	10.1	+/-	IVS 1-5 (G→C)	αα/αα
Nontribal**	17/M	4 years	4	11	4	2	4.3	8.8	62.0	20.4	4.9	77.8	10.8	+/-	IVS 1-5 (G→C)	αα/αα
Nontribal**	15/M	1 year	6	12	1	6	2.1	4.9	65.0	22.5	3.9	82.2	18.4	+/-	IVS 1-5 (G→C)	αα/αα
Nontribal**	15/M	2 years	5	10	0	3	2.7	6.7	71.0	24.5	4.0	73.1	17.3	+/-	Cd 8/9 (+G)	αα/αα

VOC = vaso-occlusive crisis; AS = asymptomatic; βthal. mut = β thalassemia mutation; NT = not tested;

* Gujarat

** Maharashtra

Most of the patients had microcytic, hypocromic anemia with low Hb, mean corpuscular volume (MCV) and mean corpuscular hemoglobin (MCH) levels. However, the mean Hb levels among the tribals were slightly lower (8.7 ± 0.60) as compared to nontribals (9.1 ± 2.04). HbF levels varied from 3 to 31.6% with a mean of 12.8%. HbF levels among the tribals were higher (18.0 ± 3.17) than the nontribals (15.1 ±; 8.20). These differences could be due to the requirement of frequent blood transfusions among the nontribals than the tribal individuals. XmnI polymorphism was studied in 19 patients of whom 13 (68.4%) were heterozygous (+/-) while only 6 (31.6%) were homozygous (+/+). HbF levels were found to be significantly higher in those who were homozygous (23.3 ± 4.48) as compared to those who were heterozygous (8.2 ± 2.63) for the XmnI polymorphism (*P* < 0.001). All the severe cases showed the presence of the XmnI polymorphism in heterozygous state (XmnI +/-), whereas 6/11 milder cases were homozygous (XmnI +/+) and the remaining 5 were heterozygous (XmnI +/-).

**Figure 1:**
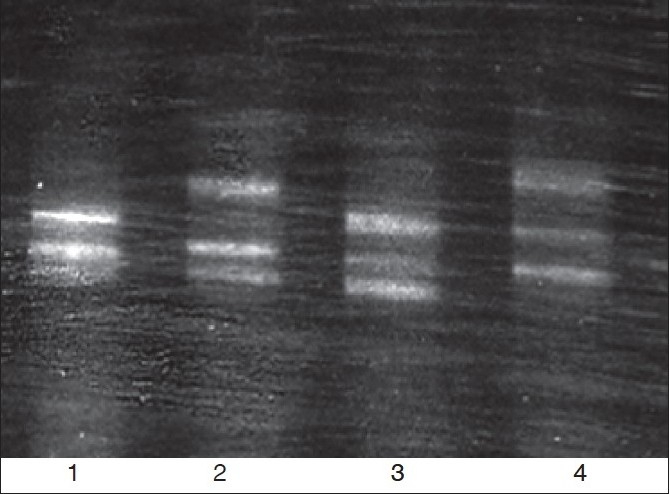
Different DGGE patterns of the codon 15 (G→A) mutation by fragment B analysis. L to R, lane 1: codon 15 (G→A) heterozygote (FW-2/2, codon 2 T/T); Lane 2: Normal control (codon 2 C/T); Lane 3: codon 15 (G→A) heterozygote (FW-3a/3a, codon 2 C/C); Lane 4: codon 15 (G→A) heterozygote (FW-1/3a, codon 2 C/T)

Four different β thalassemia alleles were seen in these cases [Table 1]. Overall, IVS 1-5 G→C was the commonest mutation which was seen only in the nontribals (52.7%), followed by codon 15 G→A (31.5%), codon 30 G→C (10.5%) and codon 8/9 +G (5.3%). All the HbS- β thalassemia cases in the tribal group had the codon 15 G→A mutation. FW analysis (fragment G of DGGE) revealed linkage of the IVS 1-5 (G→C) mutation to FW3a (FW3a), codon 30 (G→A) and codon 8/9 (+G) mutations to FW1 while the codon 15 (G→A) mutation showed three different associated framework patterns [Figure 1]. Family studies revealed that this mutation was linked to FW1, 2 and 3a. The β^s^ mutation was linked to the typical Arab-Indian haplotype (#31) in all cases.

A normal α globin genotype (αα /αα) was found in nine patients, heterozygous α^+^ thalassemia (-α /αα) in six and homozygous α^+^ thalassemia (-α /-α) in four cases, giving an α^+^ thalassemia prevalence of 52.6%. Five of the tribals screened for α thalassemia showed that all of them had either one or two α gene deletions, whereas in the nontribal group, out of the 14 cases studied, 2 were homozygous for α^+^ thalassemia (-α /-α), 3 were heterozygous (-α /αα) and 9 had a normal α globin genotype (αα /αα). All the cases showing presence of associated α thalassemia had the -α^3.7^ rightward deletion. All the severe cases had a normal α genotype (αα /αα) except one case who had one α gene deletion (-α /αα), while 9/11 milder cases were either homozygous (-α /-α) or heterozygous (-α /αα) for α thalassemia.

## Discussion

Silvestroni and Bianco[11] were the first to describe the compound heterozygosity (β^s^/β^thal^) for the sickle gene and a β thalassemia gene, and since then, HbS-β thalassemia has been reported in different ethnic groups.[4] In India, the incidence of the β^s^ gene varies from 0 to 40% and the relatively high frequency of β thalassemia in same population groups often leads to the clinically important condition, HbS-β thalassemia. The clinical and hematologic features in HbS-β thalassemia are quite variable. The clinical severity largely depends upon the nature of the β thalassemia mutations. HbS-β thalassemias are classified as HbS-β^0^ thalassemia, having absence of HbA with a severe clinical course similar to SS disease and HbS-β^+^ thalassemia usually associated with 20-30% of HbA with a milder clinical course.[12]

The four different β thalassemia mutations found in the present study were broadly similar to the distribution observed in Indians.[13] Among these, IVS 1-5 (G→C), a severe β^+^ thalassemia allele was found to be the commonest, followed by codon 15 (G→A), codon 30 (G→C) and codon 8/9 (+G) which are severe β^0^ thalassemia alleles. In the Indian population, the commonest β thalassemia mutation is seen in 30-80% of heterozygotes, while majority of the remaining β thalassemia alleles are of the β^0^ type. Earlier studies from Orissa showed that HbS-β^+^ thalassemia cases with the IVS 1-5 (G→C) mutation had low HbA levels (3–5%) similar to that observed with the IVS II-745 mutation and it had been concluded that this level of HbA does not influence the clinical expression of the disease compared to sickle cell disease in the community.[14] In the present study, codon 15 (G→A) and IVS 1-5 (G→C) were found to be the commonest mutations among the tribal and nontribals, respectively, which is similar to the earlier reports from India.[13]

Recently, it has also been shown that HbS-β^+^ thalassemia due to the IVS-1 (-2) (A→C) mutation had significant levels of HbA, indicating that this mutation is a relatively milder β^+^ thalassemia allele,[15] whereas HbS-β thalassemia involving an AT transition at codon 132 of the β-globin gene had severe clinical manifestations.[16] The effects of β thalassemia mutations on the clinical severity of HbS-β thalassemia are well documented. Perseu *et al*[17] have reported that patients with HbS-β^+^ thalassemia had a mild to moderate presentation, while those with HbS-β^0^ thalassemia showed more heterogeneity in clinical manifestations. Similarly, Schiliro *et al*.[18] and Mirabile *et al*.[19] have also shown that the type of β thalassemia alleles had an influence on the phenotypic expression of the disease. On the other hand, Nadkarni *et al*.[20] did not find any effect of β thalassemia mutations on the disease severity in the Indian thalassemic patients. Although our patients have inherited either severe β^0^ or β^+^ thalassemia mutations, the clinical presentation is quite variable and this could partially be explained by the association of α thalassemia (9/11) and XmnI polymorphism, either in homozygous or heterozygous state. Our earlier studies in sickle homozygous individuals from this region have also shown that α thalassemia is the major modulator of the severity of the disease, being more prevalent among tribals with a milder disease than among nontribals with more severe manifestations.[9]

In the present study, the IVS 1-5 (G→C), codon 30 (G→A) and codon 8/9 (+G) mutations were found to be associated with FW3a and 1, which is similar to the earlier observations from India.[10] This probably indicates a common origin of these mutations in Indian populations. The codon 15 (G→A) mutation was earlier reported to be linked with FW1 and 3a;[10] however, we observed an association of this mutation with FW2 also in a tribal individual and this raises the possibility of a new independent origin of this mutation.

All the β^s^ chromosomes in the present study were found to be linked to the typical Arab-Indian haplotype. These results are consistent with earlier reports from India[5] and further confirm the earlier observations that the origin of the β^s^ mutation in India is unicentric.[21]

We conclude that the clinical manifestations of Indian HbS-β thalassemia patients are influenced by associated α thalassemia and XmnI polymorphism rather than β thalassemia mutations.
